# 5-Year Follow-Up of a Telephone Intervention to Increase Fruit and Vegetable Consumption in Preschoolers: The ‘*Healthy Habits*’ Cluster Randomised Trial

**DOI:** 10.3390/nu12123702

**Published:** 2020-11-30

**Authors:** Rebecca Wyse, Fiona Stacey, Libby Campbell, Serene Yoong, Christophe Lecathelinais, John Wiggers, Karen Campbell, Luke Wolfenden

**Affiliations:** 1 Hunter New England Population Health, Wallsend, New South Wales 2287, Australia; fiona.stacey@health.nsw.gov.au (F.S.); Libby.Campbell@health.nsw.gov.au (L.C.); serene.yoong@health.nsw.gov.au (S.Y.); Christophe.Lecathelinais@health.nsw.gov.au (C.L.); John.Wiggers@health.nsw.gov.au (J.W.); luke.wolfenden@health.nsw.gov.au (L.W.); 2 School of Medicine and Public Health, University of Newcastle, Callaghan, NSW 2308, Australia; 3 Priority Research Centre for Health Behaviour, University of Newcastle, Callaghan, NSW 2308, Australia; 4 Hunter Medical Research Institute, New Lambton, New South Wales 2305, Australia; 5 School of Health Sciences, Swinburne University of Technology, Melbourne, VIC 3122, Australia; 6 Institute for Physical Activity and Nutrition, Deakin University, Geelong, VIC 3220, Australia; karen.campbell@deakin.edu.au

**Keywords:** fruit, vegetable, telephone-based intervention, preschoolers, long-term, cluster RCT, telephone support, parents, children’s dietary questionnaire

## Abstract

Little is known about the long-term impact of telephone-based interventions to improve child diet. This trial aimed to assess the long-term effectiveness (after 5 years) of a telephone-based parent intervention in increasing children’s fruit and vegetable consumption. Parents of 3–5 year olds were recruited from 30 Australian preschools to participate in a cluster randomised controlled trial. Intervention parents received four, weekly, 30-min support calls aimed at modifying the home food environment. Control parents received printed materials. Consumption was assessed using the Fruit and Vegetable subscale of the Children’s Dietary Questionnaire (F&V-CDQ) (children) and daily servings of fruit and vegetables (children and parents) via parent telephone interview. Of the 394 parents who completed baseline, 57% (99 intervention, 127 control) completed follow-up. After 5-years, higher intervention F&V-CDQ scores, bordering on significance, were found in complete-case (+1.1, *p* = 0.06) and sensitivity analyses (+1.1, *p* = 0.06). There was no difference in parent or child consumption of daily fruit servings. Complete-case analysis indicated significantly higher consumption of child vegetable servings (+0.5 servings; *p* = 0.02), which was not significant in sensitivity analysis (+0.5 servings; *p* = 0.10). This telephone-based parent intervention targeting the family food environment may yield promising improvements in child fruit and vegetable consumption over a 5-year period.

## 1. Introduction

Dietary risk factors are the leading preventable cause of death [1]. In 2017, low fruit and vegetable (F&V) consumption was responsible for 3.9 million deaths worldwide [2]. Internationally, population studies suggest that the majority of children consume insufficient F&V [3,4,5,6]. As greater childhood consumption of F&V is associated with a reduction in risk of cancers and stroke in adulthood [7,8], interventions to support the development of healthy dietary habits in childhood are recommended [9,10,11].

Telephone-based interventions are an accessible means of delivering dietary interventions across the population, particularly for parents of young children, as they overcome many reported barriers to engagement, including a lack of time, transport, loss of anonymity and work schedules [12,13]. Telephone-based interventions have high reach [14,15], are cost-effective [16], and an effective means of providing nutrition support to adults in disadvantaged communities [17]. As such, telephone-based services have been established in a number of jurisdictions internationally to improve public health nutrition [18,19,20].

In order for effective interventions to be of public health benefit, behaviour change needs to be sustained over a long period [21]. However, few trials of telephone-based interventions to improve diet have investigated long-term effectiveness. For example, just 10 of 25 studies in a systematic review of telephone-based interventions for physical activity and dietary change in adults investigated sustained intervention effect (i.e., 3–12 month follow-up) [22]. Only two included a dietary intervention, one of which reported that the dietary effects were maintained long-term [22]. Evidence of the long-term effectiveness of telephone-based interventions for children is particularly limited. A recent Cochrane review of interventions to improve F&V consumption of children 5 years and under identified just six studies that followed-up participants beyond 12 months, and only one was delivered by telephone [23]. This study evaluated the effectiveness of the ‘*Healthy Habits*’ intervention, conducted by the authors and the subject of this manuscript [24].

Given the effects of behavioural interventions attenuate over time [25,26] and children’s intake of F&V changes as they age [27], long-term assessment of the impact of telephone-based interventions on child diet are needed to better quantify their contribution to public health nutrition. The current study addresses this evidence-gap by conducting the longest follow-up to date of a telephone-based intervention to increase the F&V consumption of preschool-aged children (‘Healthy Habits’).

**Objectives:** The primary objective was to determine the effectiveness of a telephone-based parent intervention in increasing children’s F&V consumption after approximately 5 years. A secondary objective was to determine the effect of the intervention on parent F&V consumption.

## 2. Materials and Methods

### 2.1. Design

A full description of the trial methods has been published [28] and all previously reported assessment points were prospectively registered (ANZCTRN12609000820202). The 5-year follow-up was conducted in accordance with previously registered procedures and is reported according to the CONSORT checklist (see Table S1). The research was approved by the Human Research Ethics Committees of the University of Newcastle (H-2008-0410) and the Hunter New England Area Health Service (08/10/15/5.09). The trial used a cluster randomised controlled trial design to assess the long-term outcomes (approximately 5 years post-baseline) of the ‘*Healthy Habits*’ intervention, which commenced recruitment in February 2010. Parents of 3–5 year-old children who attended childcare services within the Hunter Region of New South Wales (NSW) Australia were randomised to receive a telephone-based intervention to increase child F&V intake (intervention) or generic print materials (control). The telephone-based intervention consisted of four, weekly, 30-min telephone support calls which increased F&V consumption as assessed by (i) the F&V subscale of the Children’s Dietary Questionnaire at 2, 6 and 12 months, and increased (ii) daily child servings of F&V at 12 and 18 months [24,29]. Outcome data were collected during parent telephone interviews at baseline and at 2-, 6-, 12-, and 18-months later [24,29] and then again approximately 5 years post-baseline (52–58 months post-intervention), which is the focus of this paper. 

### 2.2. Sample

*Childcare service recruitment*: Parents were recruited from childcare services (clusters). A random sample of all services in the region was selected and each service manager was mailed study information and consent forms. Two weeks later, a research assistant phoned the manager to confirm service eligibility and consent. To be eligible, childcare services were required not to provide children with meals (to ensure that parents had maximum opportunity to influence their children’s food consumption), not to cater exclusively for children with special needs, and not to have participated in a health promotion program targeting healthy eating in the previous 6 months. Government preschools, representing approximately 3% of services in the region, were also excluded.

*Parent recruitment:* Within each cluster, a convenience sample of parents was recruited. A research assistant, blind to group allocation, attended consenting services during either child drop-off (am) or pick-up (pm) to distribute recruitment packs and consent forms to parents. Eligible parents were required to: reside with their child at least 4 days per week; be responsible for providing meals and snacks to their child at least half of the time; and be literate in English. It was also a requirement that their child not have any allergies or dietary restrictions that would render the dietary guidelines for F&V unsuitable. Eligibility was assessed both by the research assistant at the service visit and by parents on the consent form. The research was approved by the Human Research Ethics Committees of the University of Newcastle (H-2008-0410) and the Hunter New England Area Health Service (08/10/15/5.09). The trial, with follow-ups to 18-months, was prospectively registered 21 September 2009 (ANZCTRN12609000820202). The 5-year follow-up was conducted in accordance with previously registered procedures (https://www.anzctr.org.au/Trial/Registration/TrialReview.aspx?id=320599&isReview=true).

### 2.3. Randomisation and Masking

An independent statistician randomised consenting services (clusters) in a 1:1 ratio in Microsoft Excel, using block randomisation, with block size between 2 and 6. Randomisation was stratified by the level of disadvantage of the preschool location [30]. Due to the difficulty in masking the group to which parents were allocated, this was run as an open trial.

### 2.4. Procedures

A full description of the intervention is published elsewhere [28] and is reported according to the TIDieR checklist (see Table S2).

Intervention: The intervention was based on socio-ecological theory and a family-based model of intervention [31], and consisted of written materials (a guidebook) and four 30-min weekly individual telephone support calls. Interventionists were trained telephone interviewers that had received 2-days’ basic training in the delivery of the intervention, and nutritional and parenting principles. Call content related to three domains: increasing F&V availability and accessibility, parental role-modelling of F&V consumption (given they are a critical influence on what their children eat), and introducing supportive eating routines (e.g., eating only at set meal times, at the table, without the television on) [31,32]. The intervention utilised a number of behaviour change techniques including goal setting, goal revision, self-monitoring, intention formation and using prompts or cues [33]. Table 1 maps the intervention domains and behaviour change techniques to the weekly call content. The intervention was delivered from April to December 2010.

Control: Parents were mailed a printed booklet, “The Australian Guide to Healthy Eating”, which explained the dietary guidelines and ways to meet them.

### 2.5. Data Collection

Data were collected at baseline and each follow-up via a CATI (Computer-Assisted Telephone Interview) by trained and blinded interviewers. Baseline data were collected from April-October 2010, and 5-year follow-up data from February–March 2015. At the 18-month follow-up, all participating parents were asked whether they consented to be contacted again for future follow-ups.

### 2.6. Outcomes

Primary outcome: The primary outcome was children’s consumption of F&V assessed using the F&V subscale of the Children’s Dietary Questionnaire (F&V-CDQ) [34]. A score of 14 or above indicates the child is meeting dietary guidelines for F&V consumption (range 0–28). This tool has established reliability and validity in Australian children [34], and at the time of the study, was the only such tool that was appropriate for administration via telephone. An increase in the score could arise from increases in either the variety or the frequency of F&V consumed over a 24-h or 7-day period. For example, a one-point increase could occur as a result of the child consuming an additional type of fruit or vegetable or consuming F&V at an additional occasion each day.

Other outcomes: Child—Daily servings of F&V: To indicate the quantity of F&V consumed and help contextualise the findings of the score-based F&V-CDQ, at the 12- and 18-month follow-ups two questions were added assessing the average number of F&V ‘child’ servings consumed each day (i.e., half the size of an ‘adult’ serving of fruits and vegetables). These items were adapted from questions in the Australian National Nutrition Survey, and were included on the initial consent form as well as at the 5-year follow-up. 

Parent—Daily servings of F&V: Two items from the Australian National Nutrition Survey were used to measure parents’ daily consumption of F&V at baseline and all follow-up points [35].

Demographic information: Parent age, gender, income and highest level education and identification as Aboriginal or Torres Strait Islander, and child gender and age and identification as Aboriginal or Torres Strait islander were collected at baseline. Parent income was asked again at 5-year follow-up.

### 2.7. Sample Size Calculation

The original sample size calculation indicated that 400 parents would be needed to retain 300 at 18-month follow-up, to allow a between-group detectable difference of 1.27 points on the F&V-CDQ (80% power, 0.05 significance level, assuming an ICC of 0.03) [28]. Given the 5-year follow-up was unplanned, no provision was made to increase the sample to account for parent drop-out beyond 18-months. 

### 2.8. Statistical Analysis

Parent and child demographic characteristics were analysed using descriptive statistics. To investigate potential response bias, the demographic characteristics of participants who did and did not complete the 5-year follow-up were compared within each group using Fisher’s Exact Test.

The primary outcome was assessed using a linear mixed model investigating the between-group difference in F&V-CDQ score at 5-year follow-up, controlling for baseline and potential clustering by including a random preschool intercept, as per previous follow-ups. All participants with complete data (i.e., F&V-CDQ scores both at baseline and 5-years) were included in the initial complete-case analysis. A sensitivity analysis was then conducted to assess the robustness of the initial analysis, filling in missing 5-year follow-up data through multiple imputation [36] using the SAS MI Procedure for participants that completed baseline but not 5-year follow-up. Exploratory subgroup analyses on the primary outcome (F&V-CDQ score) were conducted to determine if there were differential intervention effects among parents with higher vs. lower income and education levels, and among children who were vs. were not meeting dietary guidelines (based on baseline F&V-CDQ score) by adding an intervention group by subgroup fixed effect in each model. All other outcomes were analysed as per the primary outcome, using linear mixed models controlling for baseline and clustering.

## 3. Results

**Figure 1:**
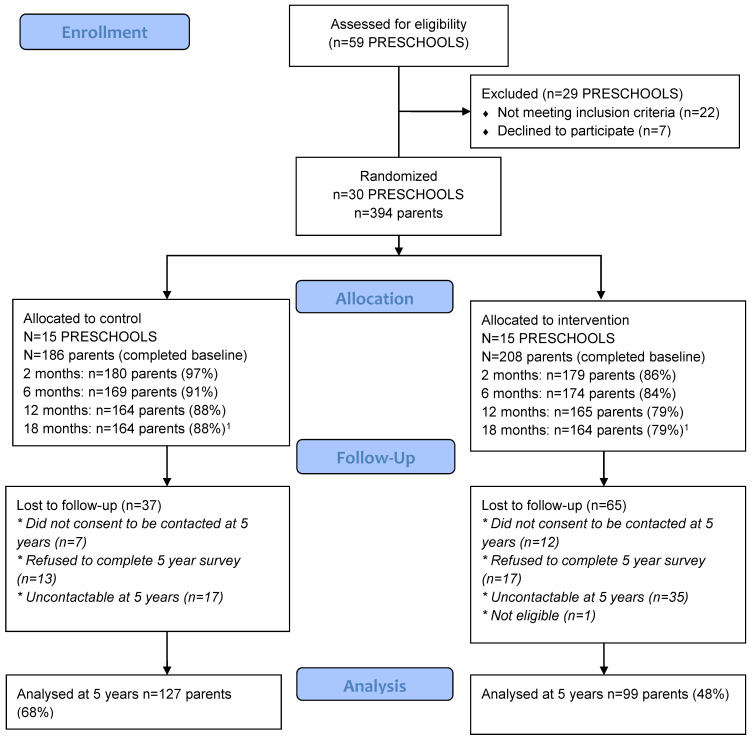
CONSORT diagram showing participant flow through trial. ^1^ Only those participants who, during the 18-month data collection call, provided consent to be subsequently contacted, were approached for the 5-year follow-up and included in these figures: Control *n* = 157, 96%; Intervention *n* = 152, 93%.

When contacted for the 5-year follow-up, 226 parents completed the interview (57% of participants that completed baseline, and 73% of those that agreed to future follow-ups at 18-month follow-up). In total, 99 intervention participants (48% of baseline participants) and 127 control participants (68% of baseline participants) completed the 5-year follow-up (chi-square *p* < 0.001). Figure 1 shows the flow of participants through the trial.

The demographic characteristics of the sample at baseline are shown in Table 2. At the 5-year follow-up, the average age of participating children was 8.5 years. The proportions of female parents (96%) and female children (51%) were similar to baseline. Compared to those who did not contribute to 5-year follow-up, participants with complete data were more likely to be university-educated (*p* = 0.007), earn ≥ AU$100,000/year (*p* = 0.03), and be older (*p* = 0.002).

### 3.1. Primary Outcome: F&V-CDQ Score

Complete-case analysis revealed higher F&V-CDQ scores for intervention children compared with control children [mean difference +1.1, 95% CI -0.03 to 2.2; *p* = 0.06], a difference that approached statistical significance. The effect was unchanged when values were imputed for missing data (*p* = 0.06) (Table 3).

### 3.2. Other Outcomes

*Child servings:* There was no significant between-group difference at follow-up in daily consumption of child fruit servings in the complete-case analysis (+0.2 ‘child’ servings, 95% CI −0.2 to 0.5; *p* = 0.26) or the sensitivity analysis (+0.1 ‘child’ servings, 95% CI -0.4 to 0.6; *p* = 0.64). There was significantly higher child intake of vegetables in the intervention group at follow-up in the complete-case analyses (+0.5 ‘child’ servings, 95% CI 0.09 to 0.9; *p* = 0.02). This effect was not significant when missing data was imputed (+0.5 ‘child’ servings, 95% CI −0.09 to 1.1, *p* = 0.10) (Table 3).

*Parent servings:* There were no statistically significant differences between parent fruit (*p* = 0.30) or vegetable (*p* = 0.27) consumption at follow-up in either the complete-case or sensitivity analysis.

### 3.3. Subgroup

There was no significant difference in the intervention group by subgroup interaction for any of the tested variables: parent education (no university education vs. university education, *p* > 0.99); household income (<AU$100,000 vs. ≥AU$100,000, *p* = 0.18); or child F&V consumption at study initiation (not meeting vs. meeting dietary guidelines, *p* = 0.50) (Table 4).

## 4. Discussion

This study reports the longest follow-up to date of a telephone-based intervention to increase F&V consumption among young children. After approximately 5 years, intervention children’s F&V consumption was higher than controls (mean difference +1.1 F&V-CDQ points) and approached statistical significance (*p* = 0.06). There was no between-group difference in parent consumption or children’s daily fruit consumption; however, there was a significant difference in daily vegetable consumption (+0.5 ‘child’ servings, *p* = 0.02). This result was not significant in the sensitivity analysis using imputed values.

The higher F&V-CDQ scores and vegetable servings among intervention children were surprising given the relatively low intensity of the four-session, telephone-based intervention, and given previous evidence suggesting intervention effects rapidly attenuate in healthy eating and telephone-based interventions [22,37]. Of the 80 studies included in a Cochrane review of interventions to increase F&V consumption in 0–5 year-olds [23], only six studies conducted follow-up of 12-months or more post-intervention and only two reported a follow-up period of 3 or more years post-intervention. The NOURISH Study followed-up 61% of participants 3.5 years after the completion of a 12-session, face-to-face intervention [38], and found that the intervention group had a higher F&V-CDQ score at follow-up (15.3 vs. 14.5, *p* = 0.03) but no effect on F&V consumption assessed by 24-h recall [38]. A similar follow-up period (3.5 years) and response rate (55%) was observed in the study by Watt et al. [39,40] which involved nine, monthly home visits conducted by trained volunteers from when the child was 10 weeks old. This study reported some significant improvements for specific F&V at 12 and 18 months [39], although it found no evidence of a long-term intervention effect on individual fruit or vegetable consumption (*p*-values ranged from 0.10 to 0.96) [40].

These interventions commenced when the children were infants, representing a key difference from the current study. Evidence suggests that dietary patterns change decisively in the preschool years, and are then more stable [41]. The children in both these studies were aged 5 years or younger at the time of long-term follow-up, whereas children in our follow-up had an average age of 8.5 years. The difference in outcomes between these studies may reflect the timing of intervention commencement and follow-up, and may have implications for selecting optimum points to provide parents with dietary support in order to ensure sustained intervention impact.

Earlier analyses found that the short-term intervention effect was mediated by parent F&V consumption and by parent provision of F&V [42]. The intervention focused on creating a home food environment that supports F&V consumption. It may be that the overt focus on environmental change may facilitate sustained changes in diet compared to individual dietary behaviour change (e.g., educational interventions). We recommend that future parent support includes strategies to improve the home food environment, and that studies investigate mediators of long-term and sustained intervention effects.

The F&V-CDQ assesses both the variety and frequency of the fruits and vegetables consumed by children. However, the individual items that measure these components have not been validated, and as such, it is not possible to determine the relative contribution of changes in variety vs. changes in frequency to the observed between-group differences in this trial. Evidence suggests that simply increasing the variety of vegetables that are served to children can increase the amount that they consume [43]. Further research is warranted to determine whether the changes in F&V-CDQ are driven mostly by changes in frequency or variety, and to determine the acceptability and feasibility of adopting strategies which target these different approaches.

Despite intervention and control participants having similar consent rates for future follow-ups at 18-months (93% vs. 96%), there was a significant between-group difference in response rates after 5-years (48% vs. 68%; *p* < 0.001). Differential attrition is common in behaviour change trials where participants cannot be blinded to study group, with a review finding 10% more attrition in intervention compared to control groups, similar to what was observed in this study [44]. A possible explanation is that intervention participants may have higher expectations in terms of intervention effectiveness, and if their expectations are not met they drop out of follow-up [44]. As such, those that contributed to 5-year follow-up may have received greater benefit from the intervention, than those who dropped-out. To counter potential bias arising from differential attrition, a multiple imputation approach was used [45] which found similar effect-sizes and significance levels to the complete-case analysis, except for child vegetable consumption (*p* = 0.10).

Despite the socioeconomic indicators of income and education being relatively similar between intervention and control groups at 5-year follow-up, it appears as though the follow-up sample had a higher proportion of university-educated parents (47.2% vs. 53.1%) and a higher proportion of households earning over $100,000 p.a. (45.7% vs. 41.4%) compared to the baseline sample (no significance testing conducted). This may indicate differential attrition over time. The subgroup analysis shows a trend towards the intervention being more effective in higher income households (between-group difference over time = 1.7 F&V-CDQ points) compared to lower income households (between group difference over time = 0.4 F&V-CDQ points). Although this difference was not significant, it is important to note that the subgroup analysis was not adequately powered. It is therefore recommended that future interventions more explicitly consider the needs of parents from lower socioeconomic backgrounds, and that long-term follow-ups employ retention strategies to reduce drop out overall, as well as among specific subgroups.

Study limitations include the use of a parent-reported food frequency questionnaire. Although a proxy measure for actual intake, validation studies suggest that parents can accurately report their child’s food consumption. For example, analysis of children’s serum levels showed that parent-completed food frequency questionnaires accurately estimate children’s usual diet over a 3-month period [46], although subsequent research suggests a more limited accurate reporting window [47]. However, the validity of the F&V-CDQ has been established in a sample of Australian children and it was found to be acceptable for assessing group level dietary patterns [34]. The representativeness of the sample is another limitation. Parents in the trial were more educated and had higher household income than average parents within the study region; and a higher proportion of children consumed the recommended daily servings of vegetables at baseline [24,29]. Furthermore, the parents that were retained for the 5-year follow-up appeared to be from higher socioeconomic backgrounds than the overall sample at baseline. The response rate is also a limitation, despite being comparable to other similar long-term studies of dietary interventions for children. Finally, the study was only powered to detect differences at the 18-month follow-up, based on a retained sample of 300 parents. The sample size calculation did not consider the attrition that would occur in a 5-year follow-up (226 parents retained, 57%), and as such was underpowered to detect differences on the primary outcome at this point. Strengths of the study include the strong research design, use of valid and reliable tools, and multiple measures of F&V outcomes in children (a score-based and serving-based measure) to triangulate evidence.

Although these results require replication, findings are encouraging. The magnitude of the long-term effect (approximately half a child serving of vegetables per day) is important at a public health level and is greater than other more intensive interventions (e.g., child feeding interventions) that governments could adopt [23]. In many jurisdictions, telephone-based preventive health services already exist (e.g., The Get Healthy Service [18], Dial-a-Dietitian [19], 8-1-1 [20]) and provide an infrastructure through which such interventions could readily be implemented and the benefits translated relatively quickly to the community. A modified version of this intervention is currently being evaluated in a patient preference trial with the NSW Ministry of Health [48], further establishing evidence for governments to use telephone-based dietary interventions to improve public health nutrition in naturalistic contexts.

## 5. Conclusions

This long-term follow-up of a telephone-based intervention to increase children’s consumption of F&V found that after 5 years, consumption was higher among intervention children and approached significance. This could reflect increases in either the variety or frequency of consumption of these foods. Furthermore, among those that contributed 5-year data, consumption of daily child servings of vegetables was significantly higher. Given the unprecedented length of follow-up for an intervention of this type, this study represents a novel contribution to the evidence-base regarding intervention sustainability. Although further research is required, this study provides preliminary evidence that telephone-based interventions, delivered in the preschool years, may represent a worthwhile investment to improve children’s F&V consumption.

## Figures and Tables

**Table 1:** Intervention content, strategies and structure [28,29].

Key Theme	Content	Behaviour Change Technique	Application of Behaviour Change Technique
WEEK 1 Availability and Accessibility	Dietary recommendations and serving sizes		
	Children’s food diary	Prompt self-monitoring of behaviour	Parents are asked to monitor their children’s intake of fruit, and vegetables over 3 days.
	Ways to provide F&V throughout the day		
	Setting goals	Prompt specific goal-setting	Parents are encouraged to set a program goal.
WEEK 2 Availability and Accessibility, Supportive Family Eating Routines	Changing the family routine	Prompt intention formation	Parents decide which activities they will attempt in the coming week.
	Availability and accessibility of foods in the home	Provide general encouragement	Interviewers provide positive feedback about any helpful practices occurring in the home.
	Mealtime practices	Teach to use prompts or cues	Parents learn the HELPS ^a^ acronym, i.e., try to eat when Hungry, not attempting anything else at the same time (focus on Eating), at an appropriate Location to eat, from a Plate, and while Sitting.
	Meal planning		
	Review of goals	Prompt review of behavioural goals	Parents review the goals they set during the previous calls and evaluate their progress.
WEEK 3 Parental role-modelling, Supportive Family Eating Routines	The Ps and Cs division of feeding responsibility	Teach to use prompts or cues	Parents learn the Ps and Cs ^b^: Parents are encouraged to Plan, Prepare and Provide. Children are encouraged to Choose (whether, what and how much to eat).
	Mealtime strategies to encourage vegetable consumption	Prompt intention formation	Parents decide which activities they will attempt in the coming week.
		Provide general encouragement	Interviewers provide positive feedback about any helpful practices occurring in the home.
	Role-modelling of fruit and vegetable consumption	Prompt identification as a role model	Parents are provided information about their importance in role-modelling fruit and vegetable consumption. Their consumption is compared with national nutrition recommendations. Tailored feedback is provided.
WEEK 4 Availability and Accessibility, Parental role-modelling, Supportive Family Eating Routines	Review of weeks 1–3	Provide general encouragement	Interviewers provide positive feedback about any helpful practices occurring in the home
	Planning for the future and dealing with difficult situations	Prompt barrier identification	Parents are encouraged to identify barriers that will prevent them implementing what they have learnt and to generate solutions.
	Review of goals	Prompt review of behavioural goals	Parents review their program goal, evaluate their progress and identify how they can maintain the change

^a^ ie, try to eat when Hungry, not attempting anything else at the same time (focus on Eating), at an appropriate Location to eat, from a Plate, and while Sitting. ^b^ Cs responsibilities of the “children”, which are to “choose whether or not to eat”, “choose what to eat from a variety of healthy options” and “choose how much to eat at scheduled meal and snack times”; Ps responsibilities of the “parents”–to “plan”, “prepare”, and “provide”.

**Table 2:** Characteristics of sample at baseline for all participants and those who completed the 5-year follow-up.

Parents Characteristics Reported at Baseline [24,29] Mean, (SD) ^a^	Baseline All Participants (*n* = 394)	Baseline Control (*n* = 186)	Baseline Intervention (*n* = 208)	5-Year Follow up All Participants (*n* = 226)	5-Year Follow up Control (*n* = 127)	5-Year Follow up Intervention (*n* = 99)
Age in years at baseline	35.5 (5.4)	35.7 (5.0)	35.2 (5.6)	36.2 (4.6)	36.2 (4.5)	36.2 (4.9)
Gender - female, (%)	95.9% (*n* = 378)	96.8% (*n* = 180)	95.2% (*n* = 198)	95.6% (*n* = 216)	96.1% (*n* = 122)	95.0% (*n* = 94)
Household income, ≥AU$100,000, (%)	41.4% (*n* = 137)	39.6% (*n* = 72)	41.0% (*n* = 84)	45.7% (*n* = 102)	44.4% (*n* = 55)	47.5% (*n* = 47)
University education, (%)	47.2% (*n* = 186)	49.5% (*n* = 92)	45.2% (*n* = 94)	53.1% (*n* = 120)	52.8% (*n* = 67)	53.5% (*n* = 53)
Aboriginal &/or Torres Strait Islander, (%)	2.0% (*n* = 8)	3.2% (*n* = 6)	1.0% (*n* = 2)	1.8% (*n* = 4)	2.4% (*n* = 3)	1.0% (*n* = 1)
Number of children <16 years	2.3 (0.8)	2.3 (0.7)	2.3 (0.8)	2.3 (0.7)	2.4 (0.7)	2.3 (0.8)
Number of fruit servings per day	1.8 (1.1)	1.8 (1.0)	1.8 (1.1)	1.9 (1.1)	1.8 (1.1)	2.0 (1.2)
Number of vegetable servings per day	3.2 (1.3)	3.1 (1.3)	3.3 (1.3)	3.2 (1.4)	3.1 (1.4)	3.3 (1.2)
CHILD CHARACTERISTICS REPORTED AT BASELINE						
Age in years at baseline	4.3 (0.6)	4.3 (0.6)	4.3 (0.6)	4.3 (0.6)	4.4 (0.6)	4.3 (0.6)
Gender—female, (%)	48.5% (*n* = 191)	45.7% (*n* = 85)	51.0% (*n* = 106)	51.3% (*n* = 116)	47.2% (*n* = 60)	56.6% (*n* = 56)
Aboriginal &/or Torres Strait Islander, (%)	2.8% (*n* = 11)	4.8% (*n* = 9)	1.0% (*n* = 2)	1.8% (*n* = 4)	1.6% (*n* = 2)	2.0% (*n* = 2)
Daily servings of fruit ^b^	2.3 (1.0)	2.2 (1.0)	2.3 (1.0)	2.3 (1.1)	2.2 (1.0)	2.3 (1.2)
Daily servings of vegetables ^b^	2.1 (1.2)	2.0 (1.2)	2.1 (1.1)	2.2 (1.1)	2.1 (1.2)	2.3 (1.1)

SD = Standard Deviation; ^a^ Unless otherwise stated; ^b^ Baseline values taken from participant consent form.

**Table 3:** Changes in child and parent fruit and vegetable consumption.

	Baseline [24,29]		5 Year Follow-up		Complete-Case ^a^: between Group Difference at 5 Years ^b^ (95% CI) *n* = 226	*p*-Value	Sensitivity analysis: between Group Difference ^b^—Imputed Values ^c^ (95% CI) *n* = 394	*p*-Value
	Control Mean (SD) *n* = 127	Intervention Mean (SD) *n* = 99	Control Mean (SD) *n* = 127	Intervention Mean (SD) *n* = 99				
Child consumption								
F&V-CDQ score ^d^	14.7 (4.6)	15.2 (4.5)	16.1 (4.4)	17.5 (3.8)	1.1 (−0.03 to 2.2)	0.06	1.1 (−0.05 to 2.3)	0.06
Daily ‘child’ servings of fruit ^e^	2.2 (1.0)	2.3 (1.2)	2.6 (1.1)	2.8 (1.3)	0.2 (−0.2 to 0.5)	0.26	0.1 (−0.4 to 0.6)	0.64
Daily ‘child’ servings of vegetables ^e^	2.1 (1.2)	2.3 (1.1)	2.9 (1.6)	3.5 (1.5)	0.5 (0.09 to 0.9)	0.02	0.5 (−0.09 to 1.1)	0.10
Parent consumption								
Daily servings of fruit	1.8 (1.1)	2.0 (1.2)	1.8 (1.5)	2.0 (1.1)	0.2 (−0.2 to 0.5)	0.30	0.3 (−0.09 to 0.6)	0.15
Daily servings of vegetables	3.1 (1.4)	3.3 (1.2)	3.5 (1.6)	3.8 (1.4)	0.2 (−0.2 to 0.6)	0.27	0.2 (−0.3 to 0.8)	0.44

CI = Confidence Interval; F&V-CDQ = Fruit and Vegetable subscale of the Children’s Dietary Questionnaire; ^a^ Includes only those participants who contributed 5-year data (i.e., complete data at baseline and 5-year follow-up); ^b^ Adjusted for baseline value of the outcome variable and clustering by preschool; ^c^ Missing 5-year follow-up data was imputed using multiple imputation; ^d^ Intraclass Correlation Co-efficient (ICC) = 0.013; ^e^ Baseline values taken from participant consent form.

**Table 4:** Changes in child fruit and vegetable consumption (F&V-CDQ): subgroup analyses.

	Baseline		5 Year Follow-up		Group by Time Differential Effect (95% CI)	*p*-Value	Group by Time by Sub-Group Differential Effect (95% CI)	*p*-Value
	Control Mean (SD) *n* = 127	Intervention Mean (SD) *n* = 99	Control Mean (SD) *n* = 127	Intervention Mean (SD) *n* = 99				
Child F&V-CDQ score								
Mtg dietary guidelines at baseline								
<14 (*n* = 82)	10.0 (3.4)	10.2 (2.9)	13.6 (5.0)	15.4 (3.9)	1.5 (−0.2 to 3.1)	0.08	−0.7 (−2.6 to 1.1)	0.50
14 or above (*n* = 144)	17.4 (2.6)	18.0 (2.4)	17.5 (3.2)	18.7 (3.3)	0.7 (−0.6 to 2.1)	0.30		
Education								
No university (*n* = 106)	13.9 (5.0)	14.3 (4.9)	15.5 (5.1)	16.9 (4.1)	1.1 (−0.4 to 2.5)	0.10	0.02 (−1.8 to 1.9)	1.0
University (*n* = 120)	15.4 (4.2)	16.0 (4.0)	16.6 (3.5)	18.1 (3.5)	1.1 (−0.3 to 2.5)	0.10		
Income								
<AU$100,000 (*n* = 121)	14.5 (4.5)	14.3 (5.0)	16.4 (4.2)	16.9 (4.1)	0.4 (−0.9 to 1.8)	0.50	−1.3 (−3.1 to 0.6)	0.18
≥AU$100,000 (*n* = 102)	14.9 (4.5)	16.2 (3.7)	15.9 (4.3)	18.2 (3.5)	1.7 (0.2 to 3.2)	0.03		

## Data Availability

Data described in the manuscript, code book, and analytic code will be made available upon request pending application and ethical approval.

## Supplementary Materials

The following are available online at https://www.mdpi.com/2072-6643/12/12/3702/s1, Table S1: CONSORT checklist; Table S2: TIDieR checklist.
